# Suicidio y prácticas médicas: la valoración del modo de vida de hombres campesinos caficultores colombianos en la atención de la salud mental

**DOI:** 10.18294/sc.2024.4663

**Published:** 2024-02-29

**Authors:** Lucy Nieto-Betancurt, Janet Mosquera-Becerra, Andrés Fandiño-Losada, Luis Alberto Suárez Guava

**Affiliations:** 1 Doctora en Salud. Profesora, Universidad Católica de Pereira; Risaralda, Colombia. lucy.nieto@correounivalle.edu.co Universidad Católica de Pereira Universidad Católica de Pereira Risaralda Colombia lucy.nieto@correounivalle.edu.co; 2 Doctora en Sociología. Profesora, Escuela de Salud Pública. Coordinadora, Maestría en Salud Pública, Universidad del Valle, Cali, Colombia. Janeth.mosquera@correounivalle.edu.co Universidad del Valle Maestría en Salud Pública Universidad del Valle Cali Colombia Janeth.mosquera@correounivalle.edu.co; 3 Doctor en Ciencias de la Salud. Profesor Asociado, Escuela de Salud Pública. Investigador, Instituto Cisalva, Universidad del Valle, Cali, Colombia. Carlos.fandino@correounivalle.edu.co Universidad del Valle Escuela de Salud Pública. Investigador Instituto Cisalva Universidad del Valle Cali Colombia Carlos.fandino@correounivalle.edu.co; 4 Doctor en Antropología. Profesor asistente, Universidad de Caldas, Manizales, Colombia. Luis.suarezg@udecaldas.edu.co Universidad de Caldas Universidad de Caldas Manizales Colombia Luis.suarezg@udecaldas.edu.co

**Keywords:** Salud Mental, Salud Rural, Suicidio, Investigación Cualitativa, Masculinidad, Colombia, Mental Health, Rural Health, Suicide, Qualitative Research, Masculinity, Colombia

## Abstract

El objetivo fue conocer el modo de vida y las prácticas de autocuidado en salud mental de los hombres adultos campesinos, que viven en un municipio del departamento de Risaralda en el eje cafetero de Colombia con alta incidencia de suicidios. Entre marzo y diciembre de 2021, se realizó un estudio etnográfico, haciendo uso de una combinación de métodos: entrevistas, observación participante, revisión documental y diario de campo. Se identificaron aspectos económicos y sociales cuyas transformaciones han afectado los roles de género, las dinámicas familiares y las posibilidades de cuidado para los hombres. Al observar cómo los hombres hablan de su sufrimiento y de los recursos con que cuentan para atenderlo, puede concluirse que las prácticas de salud mental se encuentran más bien como recursos de autoatención y los servicios de salud ofrecen atención basada en síntomas del cuerpo, de modo que abandonan la escucha. Estos hallazgos son útiles para pensar servicios y estrategias de cuidado de la vida que se adapten a las condiciones de hombres campesinos en Colombia.

## Abstract (en)

The aim was to understand the way of life and self-care practices in mental health among adult male farmers living in a municipality in the Risaralda department, located in the coffee axis of Colombia, marked by a high incidence of suicides. An ethnographic study was conducted between March and December 2021, employing a combination of methods including interviews, participant observation, document review, and field diaries. Economic and social aspects undergoing transformations were identified, impacting gender roles, family dynamics, and caregiving possibilities for these men. By observing how men discuss their suffering and the resources available to address it, it can be concluded that mental health practices function more as self-care resources, while health services often provide symptom-based care, neglecting attentive listening. These findings are valuable for shaping services and life care strategies that align with the conditions of rural men in Colombia.

## INTRODUCCIÓN

A partir del reconocimiento de la agricultura como una actividad estresante y de riesgo, la salud mental de los hombres en el ámbito rural se ha tornado un tema de interés reciente^1^^,^^2^^,^^3^^,^^4^. Diversos estudios muestran que la agricultura ha tenido cambios importantes relacionados con aspectos económicos y cómo estos afectan las formas de trabajo de quienes laboran en el campo, por ejemplo, el incremento del uso de tecnologías ha dado lugar a transformaciones sociales y, específicamente, ha impactado la interacción, lo que explica la presencia de estrés, aislamiento^3^^,^^5^, depresión^6^^,^^7^, ansiedad^6^ y muertes por suicidio^8^^,^^9^^,^^10^^)^ entre los hombres campesinos agricultores.

De hecho, las tasas de muerte por suicidio en hombres agricultores son más altas comparadas con otros grupos de hombres^8^^,^^11^ y este hecho, sumado a alta prevalencia de sintomatología y trastornos mentales en esta población^1^^,^^6^^,^^7^^,^^12^^,^^13^, ha sido el fundamento para que la atención a la salud mental sea considerada una de las estrategias orientadas a prevenir y reducir las muertes por suicidio^14^. 

Sin embargo, la aproximación a la salud mental, en general, se ha fundamentado en estudios enfocados en la presencia de sintomatología o prevalencia de trastornos mentales, al igual que cuando se trata de la salud mental de la población rural campesina^1^^,^^6^^,^^7^^,^^12^. Con relación al riesgo, se ha observado que ser hombre en el contexto rural es un punto de análisis para estimar el riesgo de suicidio y las condiciones de salud mental^9^^,^^11^^,^^13^^,^^15^. Se ha relacionado la letalidad de los intentos de suicidio con la falta de apoyo a los hombres para expresar sus sentimientos e ideaciones suicidas^16^. Aspecto también señalado por Michael Kaufman^17^ y Rita Segato^18^, al afirmar que la receptividad, la empatía y la compasión suelen ser experimentadas como inconsistentes con lo masculino.

En Latinoamérica, esta tesis se ha recogido en torno a preguntas sobre las condiciones de salud y la masculinidad, mostrando cómo la masculinidad hegemónica oprime tanto a las mujeres, como a otras masculinidades subordinadas^19^^,^^20^. Se han planteado y analizado relaciones no solo con respecto a las características individuales como la edad o el género, sino también con aspectos que van desde la consideración del acceso a la atención sanitaria, las variaciones geográficas, las crisis económicas y la manipulación de elementos letales como armas y venenos^11^^,^^15^. De otro lado, el análisis se ha orientado al miramiento de las implicaciones de las denominadas masculinidades agrícolas, en relación con cambios a nivel económico, formas de violencia y en el marco de las transformaciones culturales^9^^,^^13^^,^^19^^,^^21^^,^^22^.

Cada año, el Instituto Nacional de Medicina Legal y Ciencias Forenses (INMLCF) en Colombia presenta un informe en que se detallan los casos de violencia y las distintas formas de muerte en el territorio nacional. Se incluye una sección especial para exponer los datos sobre suicidio, acompañada de un análisis que indica la forma en que estos datos pueden ser interpretados. En los últimos años, las conclusiones de estos informes han resaltado que ser campesino, hombre y mayor de 60 años son características comunes que aumentan la vulnerabilidad para un desenlace suicida^23^^,^^24^^,^^25^^,^^26^^,^^27^^,^^28^. 

Al considerar cada una de estas condiciones identitarias como aspectos de riesgo, surge el interrogante en torno a qué hacer con un asunto que se supone de riesgo y que está ligado con la identidad. El enfoque centrado en la sintomatología y los trastornos mentales es resultado de una reducción del concepto de salud mental; es decir, se asume que la salud mental es la ausencia o el control de los trastornos mentales^29^^,^^30^. Así emerge la problemática oposición entre salud mental y suicidio: la salud mental (reducida a la ausencia de trastornos) se asume como la forma efectiva de prevenir el suicidio. Tal reducción se explica por un intento de hallar una conexión causal que permita explicar y prevenir el suicidio, con lo que se desconoce lo que sucede en la vida cotidiana de cualquier persona (fracasos, miedos, frustraciones, angustias) e incluso entre aquellos que consideran el suicidio como forma de resolver un problema^29^. 

En contraste con lo anteriormente expuesto, el estudio etnográfico realizado en una región colombiana, cuyos resultados se presentan en este artículo, pretendió prestar atención a la vida. Precisamente al modo de vida y a cómo se hace para preservar la vida, que es lo que se debería buscar en cualquier acción de cuidado de la salud y de la salud mental. El modo de vida, como una construcción teórica basal, no implica meramente conductas individuales ante la salud, sino que incluye las dimensiones sociohistóricas, la dinámica de las clases sociales y las relaciones sociales de producción, teniendo en cuenta los aspectos simbólicos de la vida cotidiana en la sociedad^31^. Por lo tanto, se trata de una categoría compleja, interactiva, inestable y dinámica^32^. 

Para comprender la salud mental en el modo de vida se precisa concebirla como prácticas sociales o sea como estrategias utilizadas para lidiar con el sufrimiento, la angustia y las necesidades de las personas y las comunidades^33^^,^^34^. Así, estudiar las prácticas de salud mental implica realizar consideraciones sobre la sociedad de mercado, pensarlas con un enfoque de derechos en el que se reflexiona la libertad, los afectados directos e indirectos, las políticas públicas, las formas de organización social, así como las amenazas colonialistas, los efectos en las poblaciones más vulnerables, los niveles de participación y la aceptación de las diferencias. De este modo, las prácticas en salud mental nos permiten ver algo más que los trastornos y los individuos, para lo que se precisa la inter y la transdisciplinariedad^35^^,^^36^^,^^37^.

De esta lectura crítica sobre el riesgo en salud mental y el suicidio, este trabajo se ocupa de develar aspectos del modo de vida y las prácticas que para cuidar su salud mental despliegan los hombres mayores campesinos que viven en el corregimiento de Peralonso en la zona rural del municipio de Santuario, que se ha indicado por la alta incidencia de muerte por suicidio. 

## MÉTODO

Se realizó un estudio etnográfico, haciendo uso de una combinación de métodos: entrevistas, observación participante, diario de campo y revisión documental.

La selección del municipio de Santuario del departamento de Risaralda se basó en los siguientes criterios: 1) según los reportes de Medicina Legal y Ciencias Forenses correspondientes al período 2014-2018^23^^,^^24^^,^^25^^,^^26^^,^^27^, la tasa promedio para Risaralda fue de 6,66, mientras que a nivel nacional fue de 4,95, por lo que Risaralda es uno de los tres departamentos, junto a Caldas y Quindío, que tuvieron las tasas de mortalidad por suicidio más altas del país; 2) a diferencia de otros municipios del departamento, como Pueblo Rico y Quinchía, el municipio de Santuario pertenece a la agrupación del Paisaje Cultural Cafetero de Colombia (PCCC), que ocupa el tercer lugar en el departamento en las denuncias por violencia de género.

El municipio de Santuario cuenta con aproximadamente 12.000 habitantes, y el 50% de su población reside en áreas rurales. Luego de entrevistar a las autoridades municipales de salud, quienes destacaron la pertinencia de incluir las zonas rurales, y tras tener en cuenta varias condiciones específicas (ubicación geográfica distante de la cabecera municipal, que dificulta el acceso a los servicios, la dinámica de la población flotante relacionada con la cosecha de café, problemas de convivencia derivados de cambios en la población y antecedentes importantes de migración y consumo problemático de sustancias), se eligió el corregimiento de Peralonso como punto de partida para adelantar el trabajo de investigación.

El trabajo etnográfico se desplegó con el uso de la observación participante^38^, la entrevista abierta^39^, el diario de campo^40^ y la revisión documental. La investigadora principal realizó una estancia de diez meses (entre marzo y diciembre de 2021) en una comunidad rural a la que llegó luego de hacer entrevistas a profesionales en la cabecera municipal. Las entrevistas fueron semiestructuradas y se realizaron a las cuatro profesionales de psicología encargadas de los servicios de salud mental para esta población, dichas entrevistas se llevaron a cabo de manera personal, en el municipio. Allí se indagó sobre la situación de salud mental, sus explicaciones e intervenciones con la población rural. 

Una vez en el corregimiento (zona rural), se entrevistaron a dos hombres y dos mujeres, quienes fueron derivados por referencia de algunos pobladores que los indicaron como líderes y lideresas, por su trabajo en la comunidad o por su edad y experiencia como caficultores en la zona. Con estos participantes se desarrollaron entrevistas semiestructuradas para abordar los temas de la vida cotidiana, el trabajo, la salud y el cuidado. A medida que la investigadora principal se fue involucrando en la vida cotidiana de las personas que habitaban el corregimiento, las entrevistas se suspendieron en su forma convencional y se incrementó la participación en actividades como mercados, reuniones de asociaciones, festejos, tareas domésticas de cuidado, y labores en torno a la tierra y la producción agrícola, que permitieron ampliar la información, sin sustraer a las personas de sus actividades para llevar a cabo la conversación. Durante este proceso de observación y participación, se adelantó la escritura del diario de campo, en el que se registraron las observaciones, los temas, las preguntas, ideas y referencias a las explicaciones que iban emergiendo de las interacciones con las personas del corregimiento. Simultáneamente, se continuó la revisión documental que se había iniciado previamente a la llegada al municipio, y se concentró en documentos relacionados con la historia de la región, el análisis de las dinámicas políticas, culturales y la transformación económica y social de la región del eje cafetero en los últimos 20 años.

Las entrevistas fueron transcritas en su totalidad y se utilizó el sotfware Atlas Ti v. 9 para realizar un proceso de codificación que da lugar a un libro de códigos y de cuyo análisis se identificaron temas iterativos que se agruparon en categorías. Las notas de campo fueron reordenadas y analizadas a través de análisis de contenido. Se codificaron y luego se analizaron de manera colaborativa entre varios de los autores y posteriormente se agruparon en categorías. El total de los datos obtenidos a través del uso de las diferentes técnicas fueron triangulados, dando lugar a los resultados del estudio.

El estudio contó con el aval ético de la Universidad del Valle, Colombia, otorgado en el acta número 154-020 de 2020. Los participantes conocieron previamente los fines del estudio y para las entrevistas firmaron un consentimiento informado. 

Este artículo es parte de un estudio más amplio sobre “Los modos de vida y las prácticas de salud mental en mujeres y hombres campesinos de poblado rural en el departamento de Risaralda”, desarrollado como trabajo de investigación doctoral. En este trabajo se abordan solo los hallazgos sobre la salud mental de los hombres del corregimiento de Peralonso.

## RESULTADOS

El corregimiento de Peralonso pertenece al municipio de Santuario en el departamento de Risaralda, Colombia. Según el reporte de la autoridad administrativa del corregimiento, para 2020 contaba con una población aproximada de 400 habitantes, de los cuales se tuvo contacto con cerca del 25% de la población durante el proceso de investigación. Se trata de un corregimiento de vocación agrícola, productora principalmente de café, plátano y frutales. Cuenta con un puesto de salud, en el que la atención se brinda los jueves cada dos semanas y se trata de consultas médicas de control y entrega de medicamentos para la atención a enfermedades crónicas no transmisibles, como la hipertensión y la diabetes. Quienes requieran servicios de salud mental deben desplazarse a la cabecera municipal que está a algo más de una hora en automóvil, con una parte del trayecto sin asfaltar (desde el corregimiento hasta la carretera principal).

La mayoría de los hombres trabajan como jornaleros (trabajadores al día en las fincas), propietarios o administradores de fincas, transporte y comercio. Las actividades de ocio y esparcimiento giran en torno a la actividad comercial, el consumo de alcohol y se despliegan, en su mayoría, en el municipio vecino de La Virginia. A continuación, se presentan los hallazgos principales relacionados con las categorías emergentes: 1) *cambios en el modo de vida: las transformaciones de las relaciones*; 2) *las relaciones con la tierra y el cuidado para los hombres: la posibilidad de hablar*; y 3) *el riesgo y el cuidado en el cuerpo de los hombres*. 

### Cambios en el modo de vida: las transformaciones de las relaciones

La caracterización de los aspectos económicos, políticos, históricos y culturales de la dinámica del modo de vida del corregimiento de Peralonso, entre los años 2011- 2020, permite reconocer aspectos característicos, como los valores centrados en la familia, el trabajo de la tierra y un liderazgo femenino caracterizado por el cuidado y la adhesión al marido. El histórico efecto de las “crisis cafeteras” trajeron consigo transformaciones en la dinámica familiar que se reflejaron en aspectos como la migración, la reconfiguración de las familias, por ejemplo, los nietos quedaron bajo el cuidado de los abuelos, mientras madres y/o padres se fueron a las ciudades capitales o salieron del país para garantizar el sustento que ya la tierra o el café no garantizaban. 

Con la declaratoria del Paisaje Cultural Cafetero Colombiano (PCCC), como proyecto de desarrollo y alternativa económica ante las “crisis cafeteras” desde el año 2011, sustentada en un discurso de unificación del territorio, se promueve el turismo como una opción de sustento económico que recoge la trayectoria caficultora y sus valores culturales, lo cual sumado a las características geográficas de la zona, promueven este territorio como un “remanso de paz”^41^. Esto ha conducido a la comercialización de un modo de vida, es decir, ese modo de vida del campesino, de la familia unida trabajando la tierra para producir café, con roles específicos para hombres y mujeres. Mientras se promueve esa imagen para atraer turistas, el modo de vida campesino transitó hacia otras formas de organización familiar y hacia nuevos y contradictorios roles.

Así, por ejemplo, la mujer debe mostrarse como líder y emprendedora, lo que relega en parte ese rol que a los hombres se ha atribuido y este último debe mostrarse apoyando el liderazgo femenino y asumir que ya no será el único proveedor, lo que ha desencadenado conflictos en la pareja y en la familia, en los que se evidencia que el modo de vida se transformó y que aquella familia comercializada es el “ideal” o una caricatura que no deja ver lo que ha cambiado. Este aspecto se hace claro en los criterios que deben cumplirse para recibir capacitaciones y mejorar las condiciones que favorezcan la obtención de un mejor precio para la venta de café, por ejemplo, si se cuenta con cédula cafetera femenina el precio por libra mejora en el mercado internacional. Sin embargo, esta representación trae para las mujeres nuevas tareas de interacción que implica cambio en el rol al salir más de casa, establecer relaciones con representantes del gremio, participar de giras que en algunos casos desatan crisis a nivel de la pareja, que pueden tener como consecuencia violencia hacia las mujeres. 

En este corregimiento, al tiempo que se gestaba un proyecto económico para los departamentos de la zona cafetera, como la declaratoria del Paisaje Cultural Cafetero Colombiano, la violencia del narcotráfico y del conflicto armado (durante mucho tiempo silenciada y que afectó tanto la zona rural como la urbana) generó desplazamientos, asesinatos^42^^,^^43^ y cambios en la perspectiva de la vida en el campo y el trabajo rural. Mientras del lado de la ilegalidad, el narcotráfico y el conflicto armado ya funcionaban como un renglón económico ante las “crisis cafeteras”, el silenciamiento que obliga la violencia, también, es requerido para comercializar el territorio por la vía del turismo. Todo esto da cuenta del cruce de formas diversas de exclusión, silenciamiento y negación: Peralonso, a pesar de reconocerse cafetero, no es reconocido como parte del Paisaje Cultural Cafetero Colombiano y, a pesar de haber sido víctima del conflicto armado, no ha sido reparado simbólicamente. 

En síntesis, el corregimiento de Peralonso se encuentra en una especie de no lugar que, a pesar de este no reconocimiento, es destino de intervenciones por parte de distintas instituciones gubernamentales y no gubernamentales, dirigidas a fortalecer la comercialización no solo del café, sino también de la oferta de servicios turísticos en torno del café. La patrimonialización, que ha traído estos servicios turísticos que se inician como proyecto de desarrollo económico, ha transformado la organización familiar, los roles de género, las formas de relación con la tierra y, en consecuencia, generan conflictos en torno a las formas de llevar la vida, siendo hombre o mujer en este campo, y la imagen que de sí debe ser proyectada para encajar en las demandas de ese mercado turístico como el de la caficultura.

En una de las conversaciones, en el marco de una reunión que tenía como fin una capacitación sobre caficultura, al saludarse, los hombres caficultores se preguntaban por qué no se habían vuelto a ver si no era con estos fines de asociación, y uno de ellos sintetizaba su experiencia en este sentido como un desarraigo y manifestó:

*Yo me siento es como un forastero. Ya uno sale y no es nadie, porque nadie lo conoce a uno y uno no conoce a nadie. Ha llegado mucha gente de afuera y los viejos o la gente con la que uno creció, o pues que es de aquí de toda la vida, ya no está. Viene uno al parque y hay pura gente nueva y ¿con quién va a hablar*? (DC0526)

Al manifestar esto, señala aspectos que no solo apuntan al cambio ocurrido en el corregimiento respecto a la dinámica comercial y su afectación en lo social, sino que denuncia cómo esto le ha llevado a experimentar una crisis de identidad, no ser nadie, no ser reconocido por nadie, y con ello una gran dificultad de no tener con quien hablar. Lo que denota este cambio en el modo de vida es el sentirse un forastero en la propia tierra, no poder contarle a nadie, “ser un viejo que no conoce a nadie”.

### Las relaciones con la tierra y el cuidado para los hombres: la posibilidad de hablar

La responsabilidad sobre la tierra y las relaciones con el cuidado constituyen una de las diferencias más importantes en los roles de género que despliegan hombres y mujeres. Así, los hombres se ocupan de la tierra que produce para comercializar, mientras que la tierra destinada a la huerta, las plantas aromáticas, medicinales y de jardín es responsabilidad de las mujeres. En el mismo sentido, unos y otros ofrecen y reciben cuidado, mientras los hombres están afuera cuidando la tierra y garantizando el ser proveedores en el sentido económico, no participan ofreciendo el cuidado a los otros miembros de la familia dentro de la casa, así la atención y la escucha como cuidado ofrecido y recibido les resulta más difícil a los hombres que a las mujeres.

Una forma en que esto puede apreciarse se observa en una reunión de la asociación que tenía como finalidad socializar un entrenamiento en la producción de cafés especiales. Antes de iniciar oficialmente la reunión, uno de los hombres que participaba se enteró de que la investigadora era psicóloga y manifestó que se sentía aburrido, como lo dijo en voz alta las personas que estaban cerca y escucharon se echaron a reír, en ese momento alzó más la voz y dijo: “¿*Por qué será que cuando uno dice que está aburrido, les da como risa, creen que uno está charlando*?”. De esta forma llama la atención no solo para decir que se siente triste, lo hace en público, reclama escucha y encuentra que no es tomado en serio. Este gesto permite interrogarse qué pasa con el cuidado que reclama este hombre, pareciera que no se puede tomar en serio la tristeza del hombre, es más común que lo haga una mujer y esto no se corresponde con lo que experimenta un hombre, y que lo diga abiertamente es motivo de risa entre los demás. Y la risa quizá no sea solo el resultado del desconcierto que produce para ellos que un hombre se atreva a referir su tristeza, sino que también puede resultar de la incapacidad de ofrecer otra respuesta, de no saber qué decir o qué ofrecer ante esa demanda, de no saber cómo cuidar de un hombre que está triste. 

Incluso una de las formas en que este hombre ha sido introducido es como alguien que “*no tiene de qué preocuparse porque tiene la platica para vivir*”, parece que solo la vía económica es válida para expresar los sentimientos, estar preocupado por los negocios es natural y factible de ser tratado por cualquier miembro de la comunidad. Sin embargo, al referirse a otras experiencias de sufrimiento, se restringe la compasión, se omiten formas de cuidado como algo que se da y se recibe, en tanto se los significa como hombres que producen, se preocupan y se encargan de proveer y es la única forma de cuidado que pueden experimentar de manera plena.

Otro hombre, a través de una de las entrevistas, revela que su proceso de envejecer y de tener que trabajar menos le ha incrementado “*la pensadera*”. Además, que como no suele compartir lo que piensa o lo que le preocupa, esta es una situación que se ha vuelto cada vez más frecuente, volver sobre hechos pasados y evaluar su comportamiento en esas situaciones, sentir arrepentimiento y la necesidad de compartirlo con alguien, lo relata así:

…*Yo tengo noches que yo me acuesto y me da por recordar todo eso, y de todas las gentes que yo conocí desde los más antiguos hasta este momento, me da por recordar todas esas gentes. Sí, eso pa’ mí es como un recordatorio, yo soy… tengo sí, por recordar, y no solo en la cama durmiendo, sino que me parece estando yo por ahí solo, ah, me acuerdo “ah, que yo que tal cosa, que yo estuve en tal parte, que yo conocí a tal parte” todo eso me da por… y recuerdo todos esos casos. De lo que me tocó que sufrir, pasar, con el uno, con el otro, todo eso sí yo lo tengo que lo recuerdo. Todo lo que me ha sucedido*. (FEJE1)

El estar por ahí sin hacer algo, o justo antes de irse a dormir es el momento que ellos (los mayores) han denominado “*la pensadera*”, “*la nostalgia*” que en otras palabras alude al sufrimiento y que antes (cuando podían exigirle más a su cuerpo) ha sido tratado poniéndose a trabajar para no pensar, de hecho, cuando un joven manifiesta estar triste o aburrido, le recomiendan que se ocupe, que se ponga a trabajar. Esta forma de evocar las experiencias pasadas y no poder comunicarlas y tampoco tener ocupación para evitarlas ha llevado también a tratar por sus medios de controlar el pensamiento, así con frecuencia entre los habitantes está el recurso de “*pedir a Dios que les cambie el pensamiento*”. En el caso de este hombre del relato, aunque los médicos le aconsejan no exigirse físicamente porque tiene un problema respiratorio, el ánimo le mejora cuando puede hacer algo por ahí en la finca, cuando se ocupa. Ahora lo ha podido compartir, ha dudado al hacerlo, durante la entrevista dice: “*usted me dio como la confianza*”. Esto es el resultado de reconocer que hay pocos espacios para la escucha, pero una vez dispuestos resulta que son aprovechados, su experiencia de escucha ha sido con frecuencia centrada en el cuerpo, en la atención médica cuando aparece como impedimento para trabajar y producir. No se puede hablar de lo que duele a los hombres si ese dolor no está en el cuerpo.

### El riesgo y el cuidado en el cuerpo de los hombres

Es con frecuencia a través del cuerpo y con comportamientos de riesgo que muchos hombres dicen e intentan sobrellevar sus situaciones de sufrimiento. Así se observa en el caso de dos jóvenes trabajadores que se suben al transporte público, que es un jeep en el que ellos van en la parte superior externa, mientras se suben, llaman la atención de todas las personas, porque hablan fuerte, están muy delgados, con cortadas en sus antebrazos, lucen sucios, y están bajo el efecto de la marihuana. Lo habitual es que estos hombres viajen en sus ropas de calle (que suelen ser las más nuevas), y que si están bajo los efectos de alguna sustancia sea el alcohol pues, aunque el uso de la marihuana es común, se realiza de modo más discreto. En cambio, estos jornaleros (como se los llama a los trabajadores que trabajan por días y van viajando de pueblo en pueblo), rompen con varias de las normas sociales de la zona, obtienen todas las miradas y la escucha no solo gracias a todas estas formas de ruptura, sino también a que cantan muy alto una canción que alude al dolor de un amor perdido, y añaden detalles de lo que hacen para enfrentar su situación expresando: “*el sábado vuelvo a La Virginia a beber para sacar esta tusa tan gonorrea*”. 

En la expresión anterior, lo que dejan saber es que van a ingerir licor, como manera de resolver ese dolor por desamor que es muy grande. Que lo harán en cuanto estén sin tener que trabajar: el día de fin de semana. Nadie en el carro los conoce, pero ya a todos han dejado saber su historia, su dolor. El primer llamado se aprecia en el abandono al que se ve sometido su cuerpo, los efectos de todos los riesgos, pero también dicen lo que están sintiendo, aunque no a alguien en particular, a cualquiera que los escuche, en cualquier lugar, sin importar o esperar una respuesta. La respuesta de los compañeros de viaje es el silencio, podría esperarse una sanción social por la ruptura de varias reglas comunes, pero lo que obtienen es un modo de atención que es respetuosa y compasiva. 

Acerca del cuidado recibido en el cuerpo, en una de las fincas uno de los hombres relata un dolor indecible por la pérdida de uno de sus hijos de manera violenta hace ya varios años. Después de esa situación, presentó varios síntomas de un trastorno de estrés postraumático (TEPT) (insomnio, ansiedad, *flash backs*), al recurrir en búsqueda de atención profesional, el cuidado ofrecido fue una “pasta” (medicamento) para tratar el insomnio. En este caso, en el momento de la atención médica, la alternativa no considera hablar de lo que le quita el sueño al hombre, sino tratar el insomnio en el cuerpo para que pudiera continuar adelantando sus actividades laborales, sin recurrir a la escucha. El hombre considera que esta prescripción hecha por el médico, al contrario de aliviarle, le causa “*enfermedad*”:

…*él me dijo que eran quesque pa que durmiera y yo no sé si sería pa los nervios o pa que durmiera, pero yo digo que eso fue como la reacción de esas pasticas porque era una pastica chiquitica así, y no me tomaba sino un solo cuartico, y yo digo que después de que me pasó como el efecto de eso fue donde ya me produció una enfermedad, eso fue una enfermedad. Me agarraron unos nervios impresionantes, yo no podía ver la gente, yo veía que pasaba una moto, o paraba pa así lejos y ahí mismo me agarraban los nervios, yo me montaba en un carro y era muerto de miedo*. (FEJE1)

Como se observa en el relato, la intervención médica no es bien valorada y como en los anteriores casos puede inferirse que este proceder responde a la idea replicada de que los hombres no quieren hablar que, como se indicó antes, también ha sido ampliamente difundida por los estudios que enfocan su atención en la salud mental de los hombres que trabajan en el campo. Pero también se corresponde con la masculinidad a la cual su rol se adecúa, el hombre que sufre, en silencio, que no dice lo que siente, que bebe para ahogar las penas, o que está bien mientras pueda trabajar. 

## DISCUSIÓN

El estudio tiene como objetivo develar aspectos del modo de vida y las prácticas que para cuidar su salud mental despliegan los hombres mayores campesinos que viven en una zona con alta incidencia de muertes por suicidio. Una de las formas en que estos hallazgos toman distancia de otras líneas de indagación previas es en el tipo de análisis del contexto que se hace posible a través del concepto de modo de vida, pues si bien se ha incrementado el volumen de trabajos que exploran la salud mental en contextos rurales, varios de estos estudios se enfocan en aspectos individuales para indicar la presencia de sintomatología asociada a trastornos mentales^6^^,^^7^^,^^44^, el suicidio^11^^,^^22^ y en el análisis de la disponibilidad y experiencia con los servicios de salud^1^^,^^45^. 

Se encuentran muy pocos estudios que, con intereses similares al nuestro, estudien aspectos del modo de vida y la salud mental en campesinos, y que hayan abordado los saberes locales y las redes sociales que los apoyan, mostrando cómo los saberes constituyen recursos sociales de salud^46^. Otros han indagado acerca de los cambios en la economía, la conexión permanente con el trabajo y la imposibilidad de separar el trabajo y la familia como aspectos importantes del cambio en la experiencia de comunidad y, por ende, de riesgo para la salud mental^5^. 

Por otro lado, es frecuente el señalamiento del cambio en las prácticas de la actividad agrícola y de cómo estas resultan en una manera importante de aislamiento o de barrera en la oportunidad de crear comunidad^2^^,^^3^^,^^5^; si bien es un aspecto que se contempla en nuestro estudio, superamos la mirada particular de la práctica agrícola, registrando las dinámicas económicas y sociales en general que se originaron en decisiones políticas regionales y en el mercado internacional del café, y cómo esto se revela también en los roles asumidos en la familia y los conflictos que esto implica en las formas de cuidado que se revelaron en las formas de relación con la tierra. 

En este sentido, al pensar las implicaciones en las formas del cuidado de la salud mental de los hombres, el aspecto más analizado en relación con el modo de vida ha sido el de la masculinidad desde enfoques interseccionales, en que se resalta la importancia de que las preguntas para el cuidado de la vida de los hombres pasen por las consideraciones de la experiencia en relación con los roles de género, los aspectos de clase, etnia/raza y plantea cómo la experiencia en relación con la salud mental es el resultado también de muchas otras formas de opresión que no pueden resolverse en el nivel individual^2^^,^^4^^,^^5^^,^^47^. En nuestros hallazgos esta diferenciación de los roles y las posibilidades de cuidado quedan abiertamente declaradas y coinciden con lo señalado en otras indagaciones que muestran cómo el hombre pensado como proveedor queda sustraído de las labores domésticas y del cuidado de los otros para mostrar su virilidad, pues debe mostrarse resistente y alguien que resiste no es vulnerable, no es cuidado por alguien más^17^^,^^20^^,^^21^. 

De esta sustracción de formas del cuidado y, en particular, sobre las prácticas de salud mental, resulta evidente en nuestros hallazgos el problema de la escisión mente-cuerpo, y cómo enfatizar el cuidado del cuerpo de los hombres y considerar las emociones como una manifestación corporal en reacción a situaciones desfavorables ha sido un favorecedor del abandono -o lo que indicamos como el énfasis en la forma de morir- revelado en la ausencia de los servicios o en su inadecuación a las necesidades de estos hombres, lo cual es un aspecto también indicado en otros estudios^1^^,^^2^^,^^45^. Nosotros podemos indicar que, efectivamente, los hombres hablan, lloran y demandan atención sobre su salud mental y esto coincide con lo hallado en agricultores de otras latitudes^3^^,^^4^^,^^9^^,^^13^^,^^15^^,^^20^^,^^21^.

Además, invita a pensar cómo está resolviéndose la atención y el cuidado disponible para ellos. En nuestros hallazgos señalamos que además de hablar acerca de lo que les ocurre, por ejemplo, debería ampliarse la exploración de eso que denominan “*los nervios*”, en tanto que el padecimiento de “*los nervios*” es unan enfermedad popular extendida por toda Latinoamérica, que aparece en muchos de los trabajos antropológicos acerca de los padecimientos populares, y que desde teorías cognitivas se plantean como corporizaciones de las relaciones individuo-sociedad, planteando la discusión en torno a la relación cuerpo/cultura^48^.

Se aprecian prácticas cotidianas que Menéndez^49^ denominaría como prácticas de autoatención. Así el trabajo, el silencio, el aislamiento, el aguante, el resistir, beber, forman parte de un proceso en el que las personas y las comunidades despliegan sus propios recursos de cuidado, para aguantar, curar, solucionar, prevenir los procesos que afectan su salud en términos reales o imaginarios. Otros estudios las han agrupado como prácticas de apoyo social y señalan cómo el hecho de encontrarse con amigos a beber café, negociar, hablar de la economía, se constituyen en una forma de comunidad y en un elemento protector para estos hombres campesinos^2^^,^^4^^,^^5^^,^^12^^,^^50^. 

Un aspecto particular de nuestros resultados apunta a considerar de manera especial los denominados “comportamientos de riesgo”, como la ingesta de bebida o el uso de drogas, pues es un asunto que constituye objeto de intervención, pero que debe continuar explorándose ante la falta de recursos de cuidado disponibles en estas condiciones del área rural y como forma activa de hacer algo con lo que les sucede. Esto implica volver a considerar la visión de riesgo que se despliega para justificar la mirada hacia estas vidas. Al indicar que ser hombre, mayor y campesino es condición de vulnerabilidad, como indica Ramírez-Ferrero^13^, podremos pensar la salud mental y contemplar la masculinidad, sus cambios y transformaciones, al ritmo del cambio en la actividad agrícola y su dinámica de mercado.

Además, es relevante analizar el papel de las y los profesionales de la salud y de servicios sociales y sus intervenciones para el cuidado, especialmente cuando lo que es riesgoso, y no deseable, contribuye a una forma de estigmatización. En este contexto, se estigmatiza la vejez, ciertas formas de masculinidad, así como la vida y el trabajo en el campo. Implícitamente, esto sugiere que ciertos modos de vida deberían cambiar o incluso desaparecer. Como se ha mencionado previamente, en los informes de Medicina Legal y Ciencias Forenses^23^^,^^24^^,^^25^^,^^26^^,^^27^^,^^28^ se alude a la salud mental como una de las necesidades de atención y el fundamento de las estrategias de intervención para prevenir los suicidios.

Como limitaciones de este estudio se señala que en las consideraciones del modo de vida debieron ampliarse aspectos relacionados con las prácticas religiosas y los líderes espirituales de la zona, que no fueron incluidas por contingencias asociadas a la ausencia de los líderes espirituales al momento de hacer el trabajo de campo.

## CONCLUSIONES

Volver a la vida de los hombres en el campo, hombres que han sido señalados en riesgo y pensar su salud mental en relación con los cambios que ha experimentado su modo de vida permite observar cómo se han afectado sus modos de relacionarse, de trabajar y, así, su identidad. En este caso, un corregimiento que ha experimentado tanto las violencias derivadas del narcotráfico como del conflicto armado, así como el tránsito de las crisis del café y con ello la transformación de la actividad agrícola y turística ha particularizado sus necesidades y prácticas de cuidado en salud mental. 

En cuanto a las prácticas de salud mental, todos estos elementos dan lugar a la identificación del tipo de cuidados que tienen a disposición y que pueden ofrecer estos hombres y cómo en buena medida están condicionados no solo por ese modo de vida, sino que particularmente se aprecia cómo los roles de género asignados por la masculinidad hegemónica juegan un papel preponderante en la experiencia de sufrimiento y en la no atención, o en otras formas que, al ser consideradas de riesgo, paradójicamente fungen como prácticas de salud mental. Estos hallazgos sobre las formas y lugares en los que los hombres hablan, y de las limitaciones de los servicios ofrecidos, posibilitan pensar y proponer alternativas de cuidado que reconozcan esos elementos de contexto y que estimen esos lugares y momentos en los que los hombres hablan para diseñar una oferta de servicios que se adapten a ellos y no que obliguen o esperen a que sean ellos quienes se adapten a los dispositivos terapéuticos institucionalmente disponibles. 

Ocuparse de la salud mental de los hombres campesinos, implica superar entonces la mirada de riesgo e identificar el rol que juegan estas formas de comportamiento, cómo eso que señalamos riesgoso es quizá una forma de manifestación y un motivo para iniciar el acompañamiento y la atención en el total sentido de la palabra. A su vez, esto implica que las intervenciones no pueden darse exclusivamente a nivel individual, sino que requiere el abordaje de aspectos culturales, sociales que son determinantes claves en la salud mental de estos hombres. 

Es así como, quizás, el horizonte de indagación al respecto debería enfocarse más en comprender estas formas de vida, donde no se trata solo de determinar probabilidades de muerte por suicidio o recursos disponibles en salud, sino asumir estas vidas como algo que está ocurriendo en el entrecruzamiento de cuestiones individuales, biológicas, sociales, económicas, políticas y culturales. La idea de considerar la prevención del suicidio en comunidades indicadas en riesgo debería prestar mayor atención a la vida (el modo de vida) y preservarla, que es lo que se busca en cualquier acción de cuidado de la salud y de la salud mental.
